# Draft Genome Sequence of the Actinomycete *Rhodococcus* sp. Strain AW25M09, Isolated from the Hadsel Fjord, Northern Norway

**DOI:** 10.1128/genomeA.00055-13

**Published:** 2013-03-07

**Authors:** Erik Hjerde, Marcin M. Pierechod, Adele K. Williamson, Gro E. K. Bjerga, Nils P. Willassen, Arne O. Smalås, Bjørn Altermark

**Affiliations:** Department of Chemistry, Faculty of Science and Technology, University of Tromsø, Tromsø, Norway

## Abstract

The cold-adapted *Rhodococcus* sp. strain AW25M09 was isolated from an Atlantic hagfish caught off the shore of northern Norway as part of an ongoing bioprospecting project that aims to identify novel bacteria with biotechnological potential. Here, we present the 5.8-Mb draft genome sequence, together with details regarding the origin of the strain and its sequence assembly.

## GENOME ANNOUNCEMENT

Members of the *Rhodococcus* genus are Gram-positive, nonmotile, nonsporulating, aerobic bacteria with a high G+C content. They have been isolated from a broad range of environments and show important potential for industrial applications (1). Here, we report the draft genome sequence of the first cold-adapted (psychrotolerant) marine member of this genus, which has been given the name *Rhodococcus* sp. strain AW25M09. The bacterium was isolated from the stomach and intestines of an Atlantic hagfish (*Myxine glutinosa*) caught in the cold waters of the Hadsel fjord, northern Norway (global positioning system [GPS] coordinates N68 30.15284, E15 00.27951), in May 2011. The animal was captured by benthic trawling at a depth of 135 m. Subsequent culturing experiments revealed that *Rhodococcus* sp. AW25M09 grows well at low temperatures in the seawater-based isolation medium IM8 (2). Phylogenetic analysis based on 16S rRNA gene sequences groups *Rhodococcus* sp. AW25M09 with four terrestrial isolates: two phytopathogens, *Rhodococcus fascians* strain DSM 20669 and *Rhodococcus cercidiphylli* strain YIM 65003 (99.3% and 99.1% identity, respectively), and two soil bacteria, *Rhodococcus yunnanensis* strain YIM 70056 and *Rhodococcus kyotonensis* strain DS472 (98.9% and 97.9% identity, respectively).

The genome of *Rhodococcus* sp. AW25M09 was sequenced using 454 sequencing technology (Roche GS FLX Titanium). The sequence reads from one shotgun library and one paired-end library (8-kb inserts) were initially assembled into 203 contigs using Newbler (3). Based on paired-end directional information, the contigs were further organized into nine scaffolds and 51 single contigs, giving a total genome size of 5.8 Mb. The largest scaffold has a G+C content of 62.4% and is approximately 5.3 Mb in size.

In all, 5,141 protein-coding genes were predicted, of which 71% could be assigned a putative function. Four copies of the rRNA genes and 46 predicted tRNAs were identified using tRNAscan-SE (4). Further analysis of the draft genome content indicated that the chromosome is circular, as determined by the absence both of terminal repeat sequences and of genes associated with chromosome linearity: *tpg*, *tap*, and *trr* (5). Three scaffolds constituting a total of ~0.2 Mb are likely to be plasmids, as they carry genes similar to those found on other *Rhodococcus* plasmids. At least seven different putative nonribosomal peptide synthetases (NRPS) are encoded in the genome, in addition to six putative polyketide synthases (PKS). The NRPS are of particular interest, as they have only 50% identity to any homolog in the NCBI nonredundant database and thus might assemble novel compounds. Additionally, 33 genes encoding putative lipases or esterases and 14 genes encoding putative proteases are present. The bacterium was found to be positive for both lipase/esterase and protease activities by plate-based functional screening using tributyrin and gelatin as substrates.

### Nucleotide sequence accession number.

This whole-genome shotgun project has been deposited at DDBJ/EMBL/GenBank under accession no. CAPS00000000.
